# Predictors of Burnout in Healthcare Workers during the COVID-19 Pandemic

**DOI:** 10.3390/healthcare9030304

**Published:** 2021-03-09

**Authors:** Adriana Cotel, Florinda Golu, Anca Pantea Stoian, Mihai Dimitriu, Bogdan Socea, Catalin Cirstoveanu, Ana Maria Davitoiu, Florentina Jacota Alexe, Bogdan Oprea

**Affiliations:** 1 Department of Psychology, University of Bucharest, 030018 București, Romania; adriana.cotel@yahoo.com (A.C.); florinda.golu@fpse.unibuc.ro (F.G.); bogdan.oprea@fpse.unibuc.ro (B.O.); 2 Department of Diabetes, Nutrition and Metabolic Diseases, Carol Davila University of Medicine and Pharmacy, 020021 Bucharest, Romania; anca.stoian@umfcd.ro; 3 Department of Obstetrics and Gynecology, Carol Davila University of Medicine and Pharmacy, 020021 Bucharest, Romania; mihai.dimitriu@umfcd.ro; 4 Department of Obstetrics and Gynecology, Sf. Pantelimon Emergency Clinical Hospital, 021659 Bucharest, Romania; falexe.jacota@gmail.com; 5 Department of Surgery, Carol Davila University of Medicine and Pharmacy, 020021 Bucharest, Romania; 6 Department of Surgery, Sf. Pantelimon Emergency Clinical Hospital, 021659 Bucharest, Romania; 7 Department of Pediatrics, Carol Davila University of Medicine and Pharmacy, 020021 Bucharest, Romania; ana.davitoiu@umfcd.ro

**Keywords:** burnout, COVID-19, health personnel, pandemics

## Abstract

The purpose of this study was to identify the predictors of burnout in healthcare workers during the COVID-19 pandemic. Data were collected from March to June in 2020, during the COVID-19 pandemic, from employees of two Romanian hospitals. Five hundred and twenty-three healthcare workers completed a series of questionnaires that measured burnout, job demands, job resources, and personal resources. Among the respondents, 14.5% had a clinical level of exhaustion (the central component of burnout). Three job demands (work–family conflict, lack of preparedness/scope of practice, emotional demands), three job resources (training, professional development, and continuing education; supervision, recognition, and feedback; autonomy and control), and one personal resource (self-efficacy) were significant predictors of burnout, explaining together 37% of the variance in healthcare workers’ burnout. Based on our results, psychological interventions during the COVID-19 pandemic for healthcare employees should focus primarily on these demands and resources.

## 1. Introduction

The outbreak of Coronavirus Disease 2019 (COVID-19) is considered a global health threat [1], becoming the third major coronavirus outbreak in recent times following severe acute respiratory syndrome (SARS) and Middle East respiratory syndrome (MERS) [2]. The challenges associated with the COVID-19 pandemic (e.g., heavy workload, work pressure, high risk of infection, inadequate resources) may affect the mental health of medical staff, such as frontline workers, mainly in terms of their burnout level [3]. Burnout represents a job-related strain as a result of repeated exposure to stressors at work, which is characterized by exhaustion (i.e., the depletion of one’s emotional and physical resources), cynicism (i.e., the negative detachment form work), and reduced efficacy (i.e., the perception of one’s lack of productivity and achievement) [4]. During the outbreak of COVID-19, the prevalence of burnout among healthcare workers ranged between 13% and 51%, depending on the country, the specific job in the hospital, and the period in which the data were collected [5,6,7,8]. However, there are insufficient data regarding the predictors of burnout during the COVID-19 pandemic. Identifying these predictors is important in order to develop the best individual and organizational interventions that could provide support for medical staff during the COVID-19 pandemic and during possible future pandemics. Since the burnout of healthcare staff is associated with decreased quality of care and decreased safety of patients [9], the efficient management of medical personnel’s burnout has practical implications for both the employees in the medical sector and for the patients, with major consequences for how the health system responds to current or future outbreaks.

Previous studies during SARS and MERS outbreaks indicate a series of job characteristics that are related to burnout in healthcare employees. After the 2003 outbreak of SARS, healthcare workers from hospitals that treated SARS patients reported higher levels of burnout than hospital employees who had not treated such patients [10]. Their perceived adequacy of training, protection, and support was negatively associated with burnout [10]. Support from supervisors, colleagues, and the organization was a negative predictor of psychiatric symptoms and of psychological distress during the SARS outbreak [11,12]. The emergency department nurses’ burnout was related to the lack of resources for treatment during the MERS outbreak [13].

These findings are in line with the Job Demands–Resources Theory (JD-R) [14], which suggests that job demands lead to a higher level of burnout and job resources decrease burnout. In addition, the theory suggests that personal resources lead to lower levels of burnout. This last assumption of the theory is supported by data from the medical sector. Under normal working conditions, personal resources such as optimism and self-efficacy are related to decreased levels of burnout in nurses [15,16].

Based on the JD-R theory and on previous research during SARS and MERS outbreaks, job demands are expected to be positively associated with burnout, and both job resources and personal resources are expected to be negatively associated with burnout in healthcare workers during the COVID-19 pandemic.

## 2. Materials and Methods

Employees of two hospitals from Romania were asked to complete a questionnaire including all the studied variables. The questionnaire was distributed in a paper-and-pencil form or in an online form. The questionnaires were distributed through the managers or the decision-makers of the hospitals. They were contacted and informed about the purpose of the study and were asked for permission to collect data. They received the hard copy questionnaires or the link to the online form and were asked to distribute them to the employees of the two hospitals. The hard copy questionnaires were collected by one of the researchers after several visits to the hospitals, following safety and protection measures. The managers or the decision-makers did not have access to the participants’ answers. The completion of the questionnaire took approximately 30 min. Data were collected from March to June in 2020, during the COVID-19 pandemic. All procedures performed in the study were in accordance with the ethical standards of the institutional research committee and with the 1964 Helsinki declaration and its later amendments or comparable ethical standards. To protect data confidentiality, participants completed questionnaires anonymously, and data were analyzed globally. The questionnaires in hard copy were stored in a safe place, and only those who performed the statistical analyses had access to the online database. Informed consent was obtained from all individual participants included in the study, according to the legal rules of informed consent [17]. Out of a total of 544 responses, 21 were invalidated due to missing values. Table 1 illustrates the characteristics of the two groups and the results of their comparison. There were no significant differences between study participants and those excluded due to missing data in relation to age, gender, tenure, and occupation.

Burnout was measured using the Maslach Burnout Inventory—General Survey [18,19]. The 16 items of the scale measure three components of burnout: exhaustion (5 items, e.g., “I feel burned out from my work.”), cynicism (5 items, e.g., “I have become less enthusiastic about my work.”), and professional efficacy (6 items, e.g., “I feel confident that I am effective at getting things done.”). The items are scored on a 7-point Likert scale, from 0 (never) to 6 (every day).

Job demands were measured with the Job Demands in Nursing Scale [20]. Lack of comfort with working conditions was measured with 4 items (e.g., “I am satisfied with my day-to-day routine”.), lack of preparedness/scope of practice was measured with 4 items (e.g., “I do not feel adequately prepared for my area of practice.”), and lack of equipment and supplies was measured with 4 items (e.g., “The equipment needed for patient care is poorly maintained”.). The instrument uses a scale from 1 (strongly disagree) to 5 (strongly agree) for all these demands. In addition, emotional demands were measured with 4 items developed specifically for health care professions [21]. Employees reported on a scale from 1 (never) to 5 (always) how often they were confronted with death, human suffering, aggressive patients, and troublesome patients. Quantitative demands were measured with 5 items (e.g., “Do you have to work very fast?”) [22] on a scale from 1 (hardly ever) to 5 (always). Finally, work–family conflict was measured with Work–Family Conflict Scale [23], composed of 5 items (e.g., “The demands of my work interfere with my home and family life”.) on a 7-point (strongly disagree–strongly agree) response scale.

Job resources were measured with the Job Resources in Nursing Scale [20]. Supervision, recognition, and feedback was measured with 4 items (e.g., “I feel validated by my supervisor for a job well done”.), training, professional development, and continuing education was measured with 4 items (e.g., “I am able to access an adequate number of in-services or continuing education activities”.), staffing and time was measured with 4 items (e.g., “There are enough staff members in my work setting to get the job done”.), technology was measured with 4 items (e.g., “I am able to provide better care because of the information systems and technology available to me”.), autonomy and control was measured with 4 items (e.g., “My job description is flexible; I am able to modify my daily duties or the type of work that I do”.). The instrument uses a scale from 1 (strongly disagree) to 5 (strongly agree) for all these resources. In addition, social support was measured with the Job Demands–Resources Questionnaire [24], using three items (e.g., “If necessary, can you ask your colleagues for help?”) on a scale from 1 (never) to 5 (very often).

Personal resources were measured with the Job Demands–Resources Questionnaire [24]. Self-efficacy (e.g., “I can handle whatever comes my way”.) and optimism (e.g., “I usually expect the best in uncertain times”.) were measured with four items each on a scale from 1 (absolutely wrong) to 4 (absolutely right).

## 3. Results

Burnout scores ranged from 0 to 5, *M* = 1.35, *SD* = 0.93; exhaustion scores ranged from 0 to 6, *M* = 2.05, *SD* = 1.31; cynicism scores ranged from 0 to 5.60, *M* = 1.27, *SD* = 1.10; professional inefficacy scores ranged from 0 to 5, *M* = 0.94, *SD* = 0.92. Statistical analyses indicated an adequate reliability for the burnout measurement: Cronbach’s *α* = 0.89 for the overall score, Cronbach’s *α* = 0.84 for exhaustion, Cronbach’s *α* = 0.74 for cynicism, and Cronbach’s *α* = 0.80 for professional inefficacy. We tested the factor structure of the burnout measure by conducting a series of confirmatory factor analyses using M plus 7.0 [25] in order to investigate the validity of the hypothesized measurement model. The first-order and second-order theoretical models of burnout were compared with the model in which all items loaded on a single factor. The fit indices (χ^2^ = 295.64, df = 99, χ^2^/df = 2.99, CFI (Comparative Fit Index) = 0.94, TLI (Tucker–Lewis Index) = 0.93, RMSEA (Root Mean Square Error of Approximation) = 0.06 (CI = 0.05, 0.07), SRMR (Standardized Root Mean Square Residual) = 0.05) for the first-order model (in which the three components of burnout were loaded by their specific items) showed a good fit with the data. Similar results (χ^2^ = 295.64, df = 99, χ^2^/df = 2.99, CFI = 0.94, TLI = 0.93, RMSEA = 0.06 (CI = 0.05, 0.07), SRMR = 0.05) were found for the second-order model (in which a general burnout factor was loaded by exhaustion, cynicism, and professional inefficacy, which in turn were loaded by their specific items). An alternative model, in which all items loaded on a single factor, showed poor fit with the data (χ^2^ = 639.60, df = 102, χ^2^/df = 6.27, CFI = 0.84, TLI = 0.81, RMSEA = 0.10 (CI = 0.09, 0.11), SRMR = 0.07). The burnout measure showed a good fit because CFI (Comparative Fit Index) and TLI (Tucker–Lewis Index) were above 0.90 [26], the RMSEA (Root Mean Square Error of Approximation) was 0.06, and the SRMR (Standardized Root Mean Square Residual) was lower than 0.08 [27]. Therefore, the burnout measure adopted in our study was valid. Since the cutoff points presented in the Dutch version of the Maslach Burnout Inventory (MBI) manual or recommended in other studies were not able to satisfactorily differentiate between clinical and non-clinical burnout, we used a 3.50 cutoff point for exhaustion in order to minimize false negatives [28]. Based on this cutoff point, 76 (14.5%) of the healthcare workers had a clinical level of exhaustion during the COVID-19 pandemic in Romania. There were no differences between men and women in terms of their burnout level (*p* > 0.05). Additionally, there were no differences between professions regarding the level of burnout (*p* > 0.05).

Table 2 shows the means, standard deviations, reliabilities, and the correlations with burnout (the overall score and the three factors) for the variables included in the study (job demands, job resources, and personal resources). As expected, job demands were positively associated with burnout, and both job and personal resources were negatively associated with burnout.

A three-stage hierarchical multiple regression was conducted in order to predict healthcare workers’ burnout during the COVID-19 pandemic based on their job demands, job resources, and personal resources. All the job demands were entered at stage one. A significant regression equation was found; *F*_(6, 516)_ = 27.128, *p* < 0.001, with an *R*^2^ of 0.23. Job demands accounted for 23% of the variation in burnout. All the job resources were entered at stage two. A significant regression equation was found; *F*_(12, 510)_ = 20.074, *p* < 0.001, with an *R*^2^ of 0.31. Adding job resources to the regression model explained an additional 8% of the variation in burnout during the COVID-19 pandemic. The final model also included personal resources; *F*_(14, 508)_ = 22.487, *p* < 0.001, explaining an additional 6% of the variation in burnout. The final model explained 37% of the variance in healthcare workers’ burnout. When all the independent variables were included in the regression model, only three job demands (work–family conflict, lack of preparedness/scope of practice, emotional demands), three job resources (training, professional development, and continuing education; supervision, recognition, and feedback; autonomy and control), and one personal resource (self-efficacy) were significant predictors of burnout in healthcare workers during the COVID-19 pandemic. The regression statistics are presented in Table 3. The results are in line with our hypotheses.

## 4. Discussion

The results of this study are in line with the JD-R theory [14] and with previous research during SARS and MERS outbreaks. Job demands were positively associated with burnout during the COVID-19 pandemic, as in the case of the MERS outbreak for emergency department nurses [13]. A negative relationship between job resources and burnout was found in both the current study and during the SARS outbreak, when training, protection, and support from supervisors, colleagues, and the organization were negative predictors of psychological stress and burnout [10,11,12]. Regarding the negative relationship between personal resources and burnout, our results are in accordance with findings under normal working conditions in healthcare [15,16]. Workplace stressors for medical staff has been studied in Romania before but not in pandemic conditions [29]. As far as we know, only one study investigated the burnout of Romanian medical personnel during the pandemic, but it focused only on prevalence [30]. The present study contributes to the development of knowledge related to burnout in the medical field during pandemics by highlighting a number of predictors.

Based on our results, psychological interventions during the COVID-19 pandemic for healthcare employees should focus primarily on three job demands (work–family conflict, lack of preparedness/scope of practice, emotional demands), three job resources (training, professional development, and continuing education; supervision, recognition, and feedback; autonomy and control), and one personal resource (self-efficacy). The existing data support the efficiency of some interventions in reducing burnout. Three types of interventions that reduce exhaustion have been identified: those based on relaxation techniques, those that provide new role-related knowledge and work skills, and those that provide cognitive-behavioral therapy [31]. Moreover, job crafting interventions have a positive effect on the well-being and performance of employees in the medical sector [32]. Finally, self-efficacy can be increased with psychological capital interventions [33]. These types of interventions can be used in order to reduce the effect of the identified predictors on burnout.

This study has a number of limitations. Firstly, the job characteristics during the COVID-19 pandemic were measured with self-report instruments. The collected data do not provide an objective evaluation of actual demands such as lack of preparedness or resources such as supervision. Secondly, the sample consists of Romanian employees, raising concerns regarding the generalizability of our findings to other countries. Finally, the study was cross-sectional; therefore, we cannot draw causal conclusions. Future longitudinal studies could identify predictors of medical staff burnout in other countries and using multiple measurement methods.

## 5. Conclusions

This paper contributes to the field by extending the JD-R model’s assumptions about predictors of burnout in particular work situations, such as the context of an outbreak for healthcare workers. In line with the model, burnout was associated with high demands and with the lack of job and personal resources, supporting the utility of JD-R in understanding negative psychological states at work during pandemics. Our findings suggest that psychological interventions during the COVID-19 pandemic for healthcare employees should focus primarily on three job demands (work–family conflict, lack of preparedness/scope of practice, emotional demands), three job resources (training, professional development, and continuing education; supervision, recognition, and feedback; autonomy and control) and one personal resource (self-efficacy). In these demanding circumstances, practitioners in the field of occupational health psychology can implement cognitive-behavioral interventions, relaxation techniques, job crafting interventions, psychological capital interventions, and trainings aimed at developing work-related knowledge and skills.

## Figures and Tables

**Table 1:** Comparison between included and excluded participants.

Variables	Included Sample (*n* = 523)	Excluded Sample Due to Missing Values (*n* = 21)	Test
Age (years) (*M*/*SD*)	42.86/9.43	43.53/12.03	*t* = −0.30, *p* = 0.77
Gender	19% men	19% men	χ^2^ = 0.00, *p* = 0.99
	81% women	81% women	
Job tenure	5% under 1 year	5% under 1 year	χ^2^ = 3.25, *p* = 0.51
	8% between 1 and 3 years	0% between 1 and 3 years	
	15% between 3 and 5 years	15% between 3 and 5 years	
	7% between 5 and 10 years	15% between 5 and 10 years	
	65% over 10 years	65% over 10 years	
Occupations	28% physicians	14% physicians	χ^2^ = 3.54, *p* = 0.17
	67% nurses	86% nurses	
	5% other occupation (e.g., stretcher-bearers)	0% other occupation	

**Table 2:** Means, standard deviations, reliabilities, and correlations with burnout (*n* = 523).

Variables	*M*	*SD*	*α*	Burnout	Exhaustion	Cynicism	Professional Inefficacy
*Job demands*							
Emotional demands	3.31	0.70	0.59	0.23 ***	0.24 ***	0.19 ***	0.14 **
Quantitative demands	3.43	0.74	0.65	0.25 ***	0.35 ***	0.14 **	0.12 **
Work–family conflict	3.71	1.63	0.93	0.30 ***	0.39 ***	0.18 ***	0.17 ***
Lack of comfort with working conditions	2.79	0.84	0.76	0.22 ***	0.34 ***	0.13 **	0.08
Lack of preparedness/scope of practice	1.56	0.60	0.68	0.28 ***	0.15 ***	0.26 ***	0.32 ***
Lack of equipment and supplies	2.57	0.92	0.75	0.28 ***	0.28 ***	0.24 ***	0.17 ***
*Job resources*							
Autonomy and control	3.20	0.77	0.62	−0.34 ***	−0.35 ***	−0.27 ***	−0.26 ***
Supervision, recognition, and feedback	3.27	0.92	0.80	−0.36 ***	−0.33 ***	−0.31 ***	−0.32 ***
Training, professional development, and continuing education	3.41	0.87	0.78	−0.40 ***	−0.35 ***	−0.36 ***	−0.32 ***
Staffing and time	2.89	0.90	0.73	−0.26 ***	−0.37 ***	−0.15 ***	−0.12 **
Technology	3.41	0.67	0.72	−0.27 ***	−0.24 ***	−0.23 ***	−0.25 ***
Social support	3.76	0.93	0.75	−0.15 ***	−0.06	−0.17 ***	−0.20 ***
*Personal resources*							
Self-efficacy	3.51	0.56	0.88	−0.39 ***	−0.31 ***	−0.31 ***	−0.35 ***
Optimism	3.90	0.88	0.89	−0.30 ***	−0.23 ***	−0.28 ***	−0.22 ***

Footnotes: *M* = mean, *SD* = standard deviation, *α* = Cronbach’s alpha, ** *p* < 0.01, *** *p* < 0.001.

**Table 3:** Summary of hierarchical regression analysis for variables predicting burnout (*n* = 523).

Variables	Model 1			Model 2			Model 3		
	*B*	*SE B*	*β*	*B*	*SE B*	*β*	*B*	*SE B*	*β*
Work–family conflict	0.10	0.03	0.18 ***	0.09	0.03	0.16 ***	0.07	0.03	0.12 **
Lack of equipment and supplies	0.13	0.04	0.13 **	0.05	0.04	0.05	0.01	0.04	0.01
Lack of preparedness/scope of practice	0.40	0.06	0.26 ***	0.32	0.06	0.21 ***	0.16	0.06	0.10 **
Quantitative demands	0.07	0.06	0.05	0.00	0.06	0.00	−0.01	0.06	−0.01
Emotional demands	0.22	0.06	0.17 ***	0.18	0.05	0.14 **	0.21	0.05	0.16 ***
Lack of comfort with working conditions	0.11	0.05	0.10*	0.07	0.05	0.06	0.05	0.04	0.05
Training, professional development, and continuing education				−0.16	0.05	−0.15 **	−0.15	0.05	−0.14 **
Supervision, recognition, and feedback				−0.13	0.05	−0.13 **	−0.15	0.04	−0.15 ***
Autonomy and control				−0.13	0.05	−0.11 *	−0.11	0.05	−0.09 *
Technology				−0.04	0.06	−0.03	−0.04	0.06	−0.03
Staffing and time				0.03	0.05	0.03	0.03	0.05	0.03
Social support				−0.07	0.04	−0.07	−0.07	0.04	−0.07
Self-efficacy							−0.43	0.04	−0.26 ***
Optimism							−0.06	0.07	−0.06
*R^2^*	0.23			0.31			0.37		
*F* for change in *R^2^*	27.13 ***			10.14 ***			25.43***		

Table footnotes: * *p* < 0.05, ** *p* < 0.01, *** *p* < 0.00.

## Data Availability

The data presented in this study are available on request from the corresponding author. The data are not publicly available due to confidentiality reasons.
